# Horner's Syndrome Secondary to Epidural Anesthesia After Scoliosis Correction: A Case Report

**DOI:** 10.7759/cureus.20071

**Published:** 2021-12-01

**Authors:** Ali Atoot, Monica Paganessi, Michael Block, Mark D Schlesinger

**Affiliations:** 1 Anesthesiology, Hackensack University Medical Center, Hackensack, USA

**Keywords:** horner’s syndrome after epidural, pediatric horner’s syndrome, scoliosis correction, epidural anesthesia, horner's syndrome

## Abstract

Horner’s syndrome is a clinical triad composed of ptosis, miosis, and facial anhidrosis. Overall, this complication is rare but well-documented in the obstetric population receiving labor epidural analgesia, which usually follows a relatively benign transient course. Less commonly, few cases of Horner’s syndrome have been reported in the pediatric population following surgical correction of scoliosis with epidural placement. We present a rare case of a pediatric patient that developed Horner’s syndrome secondary to epidural anesthesia following surgical correction of scoliosis.

## Introduction

Horner’s syndrome is caused by disruption of the sympathetic outflow to the ipsilateral head and neck involving the face, pupil, levator palpebrae, and conjunctiva. A rare complication of epidural analgesia, the development of Horner’s syndrome is most commonly seen in women during labor or cesarean section [1]. The reported incidence is thought to range up to 4% within this population of parturient patients [2]. Horner’s syndrome as a complication after scoliosis correction in the pediatric population is rare, and to our knowledge, there are only two reported cases. We present a rare case of a pediatric patient that developed Horner’s syndrome secondary to epidural anesthesia following surgical correction of scoliosis.

## Case presentation

A 12-year-old girl underwent posterior correction for adolescent idiopathic scoliosis with a thoracic curve in the mid-40 degrees, a lumbar curve at 72 degrees, and a large decompensated left-sided trunk shift. Preoperative pediatric orthopedic and anesthetic assessments revealed no comorbidities. A posterior instrumented fusion was completed under general anesthesia. Direct laryngoscopy was performed using a size 6.0 endotracheal tube. Propofol, fentanyl, and midazolam were used for induction. Peripheral access, arterial line, indwelling urinary catheter, and leads for motor and sensory-evoked potentials were placed. The eyes were taped closed and protected, free of pressure, and rechecked frequently. The patient was placed prone onto a Jackson table with chest and hip padding. The patient’s cervical spine was maintained in a neutral position with shoulders secured in abduction. Pressure points were protected and checked throughout. General anesthesia was maintained for approximately six hours Systolic blood pressure was maintained between 80-130. Warming devices were used to maintain normothermia throughout. The surgical course was uncomplicated. Spinal cord monitoring (motor/somatosensory-evoked potential) was normal throughout. Good correction was achieved.

Two epidural catheters were introduced into the T7-T8 osteotomy site. One catheter advanced cephalad extended to T5 and one advanced caudad extended down to T12. No blood or cerebrospinal fluid was aspirated. Extubation and immediate recovery were uncomplicated. Bupivacaine (0.1%) and fentanyl (2 μg/mL) were delivered for postoperative analgesia through both catheters. The cephalad epidural had a continuous infusion rate of 4 mL/hr with no demand doses and a maximum hourly volume of 4 mL. The caudad epidural had a continuous infusion rate of 6 mL/hr, a demand dose of 2 mL with lockout every 20 minutes, and a maximum hourly volume of 12 mL. The pain was well controlled, with a pain score of 2/10.

On postop day one, as the patient was ambulating to the bathroom, the caudad epidural was noted to have been dislodged and removed. The cephalad epidural remained in place at its proper position, with no movement noted at the skin marking after initial placement. Dosing for the remaining catheter remained the same. The patient remained stable for the remainder of the day. On postop day 2 (41 hours post-op), unequal pupils were noted and measured 2 mm (right) and 4 mm (left) with normal light reflexes. The full neurological exam was otherwise normal. Motor and sensory block assessment remained unchanged from prior. The patient was hemodynamically stable with no associated headache or visual or psychological disturbances. Based on these clinical findings, a diagnosis of Horner’s syndrome was made by the primary care team. At this time, the remaining cephalad epidural was discontinued and removed with the distal tip intact. Four hours later, Horner’s syndrome signs and symptoms were completely resolved with no residual deficit. The patient received multimodal analgesia as needed for pain. No additional follow-up was required for Horner's syndrome. The patient was discharged with instructions for routine postoperative follow-up with her primary care physician.

## Discussion

Horner’s syndrome is caused by the disruption of the oculosympathetic pathway, affecting ipsilateral sympathetic innervation to the face, pupil, levator palpebrae, and conjunctiva [3]. These sympathetic preganglionic B fibers originate from the anterior horn cells from C8-T2, occasionally as low as T4, as second-order neurons that terminate in the superior cervical ganglion [4]. These fibers are small and myelinated, thus possessing higher sensitivity to low concentrations of local anesthetics [5-6].

A rare complication of epidural analgesia, the development of Horner’s syndrome is most commonly seen in women during labor [1-2]. Horner’s syndrome with epidural anesthesia is more likely to occur during pregnancy, likely due to the anatomical and physiological changes favoring high cephalad spread of local anesthetics that are injected into the epidural space [7]. The gravid uterus results in increased intra-abdominal pressures and partial obstruction of the inferior vena cava, thus diverting blood through the epidural venous plexus resulting in epidural venous engorgement and reducing volume within the epidural space [8-10].

To our knowledge, there have only been two prior reported cases of Horner’s syndrome occurring after posterior spinal fusion with the use of epidural analgesia [11-12]. Horner’s syndrome related to epidural analgesia usually follows a relatively benign course with complete resolution of symptoms within a few hours. For patients developing symptoms following epidural analgesia for labor, the mean duration was 215 minutes [8]. Hered et al. described a case in which the pediatric patient developed persistent Horner’s syndrome that eventually required surgical repair of the ptosis six months after presentation [12]. We also note a case report by Cowie et al. who described complete symptomatic resolution within 14 hours of onset in a pediatric patient after scoliosis correction with the use of epidural analgesia [11]. As was observed in our reported case, the patient developed symptoms of Horner’s syndrome approximately 41 hours postoperatively with miosis in the right pupil without any other additional signs of paresthesia and hemodynamic or respiratory compromise. Fortunately, our patient had complete symptomatic resolution within just a few hours.

Similar to the mechanism proposed in parturient patients described above, in uncorrected scoliosis, the width of the epidural space differs depending on the vertebral level and usually favors the convex side [13]. The abnormal narrowing along the epidural space in patients with scoliosis may give rise to increased epidural pressures leading to potential complications including Horner’s syndrome. However, given that epidural analgesia was not started until after the scoliosis correction, in this case, the described mechanism is a less likely explanation. The most likely explanation of transient Horner’s syndrome in our patient is the increased sensitivity of the sympathetic nerve supply to local anesthetics.

## Conclusions

We have presented a detailed case of transient Horner’s syndrome developing in a pediatric patient who underwent scoliosis correction with the use of epidural analgesia for postoperative pain management. Horner’s syndrome in the setting of epidural analgesia is a rare complication that is seen more often in obstetric patients undergoing labor; however, it can be seen within the pediatric population as well. Although this complication is very rare and follows a relatively benign and self-limiting course, clinicians should be mindful that serious and life-threatening adverse outcomes are possible. In our care, the quick recognition allowed us to cease the transfusion, remove the catheter, and continue multimodal analgesia for pain control with no further complications.
